# White Coats Under Fire: Understanding the Epidemic of Violence Against Doctors

**DOI:** 10.7759/cureus.66346

**Published:** 2024-08-07

**Authors:** Satvik N Pai, Madhan Jeyaraman, Naveen Jeyaraman, Sankalp Yadav

**Affiliations:** 1 Orthopaedics, PESU Institute of Medical Sciences and Research, Bengaluru, IND; 2 Orthopaedics, South Texas Orthopaedic Research Institute, Laredo, USA; 3 Clinical Research, Viriginia Tech India, Chennai, IND; 4 Orthopaedics, ACS Medical College and Hospital, Dr. M. G. R. Educational and Research Institute, Chennai, IND; 5 Medicine, Shri Madan Lal Khurana Chest Clinic, New Delhi, IND

**Keywords:** causes, healthcare workers, doctors, violence, violence against doctors

## Abstract

The doctor-patient relationship, once grounded in trust and mutual respect, is increasingly marred by incidents of violence against healthcare providers. This alarming trend not only threatens the safety of doctors but also undermines the integrity of medical care. This article delves into the multifaceted reasons behind such violence, exploring emotional, financial, societal, and systemic factors that contribute to this disturbing phenomenon. Drawing from extensive surveys and real-world incidents, we shed light on the pressures and misunderstandings that fuel hostility towards medical professionals. Our analysis identifies key stressors, including heightened emotions, lack of understanding, financial burdens, societal prejudices, and systemic frustrations, which exacerbate tensions in healthcare settings. By understanding these underlying causes, we offer practical recommendations for doctors to navigate these challenges, emphasizing empathy, clear communication, and professional boundaries. Additionally, we highlight the need for systemic reforms, such as better security measures in hospitals and effective grievance redressal systems, to protect doctors and improve the overall healthcare environment. This article aims to raise awareness, foster dialogue, and provide actionable solutions to mitigate violence against doctors, ultimately striving to restore the sanctity of the doctor-patient relationship.

## Editorial

The doctor-patient relationship is unique in many ways. It is one between a client and service provider but it is so much more than that in so many ways. Trust, ethics and gratitude are integral pillars of that relationship. That relationship has changed over the past few decades. Nothing exemplifies the change in that relationship more than the rising incidents of violence against doctors and other healthcare workers. This violence may be in the form of psychological or physical violence, both being equally destructive to the sanctity of the doctor-patient relationship. In this article, we will do a detailed analysis of the reasons why patients or patient attendees resort to violence against healthcare professionals. Based on these aspects we identify, we recommend certain changes in the behaviour of doctors that would help to alleviate or work around these problem points. No doubt that the legal protection of doctors and strong punishments for violence are important, but here we explore other measures that may be more under the control of doctors.

How bad is the problem?

According to a survey conducted by the Indian Medical Association in 2015, more than 75% of doctors across the country have faced violence in some form, at least once during their career [1]. The problem is not restricted to India though. As per data released by the World Health Organisation in 2020, the reported range of physical violence faced by health workers is estimated to be somewhere between 8% and 38% [2]. In India, the numbers seem to be higher than the global averages. A single-year statistics analyzing incidents of violence against doctors reported in 10 leading newspapers found 93 reported incidents of physical violence against doctors, with Maharashtra and Karnataka being the states with the highest reported cases [3]. Among the reported physical violence against healthcare workers, 81% were against doctors, 6.5% on staff nurses, and 13% were faced by other health personnel. This, however, is only the numbers which have gained media attention and is the tip of the iceberg. The numbers at the ground level are far larger. A survey conducted in a government hospital in Uttar Pradesh found that 69.5% of doctors reported having experienced violence in one or another form in just the past year [2]. A survey conducted among doctors across the country found the number to be at 77.3% of doctors having ever faced workplace violence [4]. Just over the past year, there have been multiple incidents of violence against doctors, including incidents where the doctors have unfortunately lost their lives. In January 2023, two doctors of the Vasantrao Naik Government Medical College in Maharashtra's Yavatmal were assaulted and stabbed by a patient. In May 2023, Dr. Vandana Das, a house surgeon working in Kottarakkara Taluk Hospital in Kollam, Kerala, was stabbed to death by a patient.

Understanding the reasons

To understand the reasons behind this worrying trend, we must delve deeper into the cause of this problem. Only if we understand what the root cause is can we begin to effectively manage the situation. While law and the fear of punishment should provide a very strong deterrent to such acts of violence, we also want to identify the motivations behind such attacks, so that they can be eliminated at the source. Some studies have tried to investigate this with basic surveys. A survey of 617 doctors in India found that ‘actual or perceived non-improvement or deterioration of patient's condition’ - 40%, followed by ‘perception of wrong treatment given’ - 37% were the main causes of workplace violence [4]. Family members/relatives were the major perpetrators in 82.2% of the cases. While those are precipitating factors in most of the incidents, we also wanted to explore deeper problems in the doctor-patient dynamic that lead to such incidents.

‘No physician, however conscientious or careful, can tell what day or hour he may not be the object of some undeserved attack, malicious accusation, blackmail or suit for damages’. You would think that is a quote following the recent rise in violence against doctors. It isn’t. It is a quote from an article published in the *Journal of American Medical Association* over 135 years ago [5]. The fact that this was a concern even to the medical men a century ago is reflective of our belief that certain aspects of the doctor-patient relationship may inherently be plagued with this problem, irrespective of the recent trends. We have identified eight factors which we believe are putting the doctor-patient relationship on its collision course. The first three factors we believe are the predominant factors, with the other five factors also playing contributory roles. We delve deep into each of these aspects to understand its origin and implications. Based on that understanding, we recommend how these factors can be tackled by doctors to some extent (Table 1).

**Table 1:** Recommendations for the factors influencing violence.

Factors influencing violence	Significance	Recommendation
Emotion	Unlike other client-service provider relationships, emotion is a much bigger factor in the doctor-patient relationship. Understandably so, patients and their close ones are in an emotionally vulnerable state when one’s health is deteriorating or in the balance. When an individual has a health problem and is admitted to the hospital, it is probably one of the toughest times for an individual emotionally, and similarly for the close ones of that individual. Hospitals would probably be rated highest amongst the public locations of heightened emotions of anxiety and sorrow. In this emotionally charged situation, individuals are more likely to have an emotional outburst. While it is preferable to have that in terms of an expression of feelings, some individuals who are unable to control their emotions may resort to other expressions of anger and sadness in the form of violence.	We as doctors must be understanding of the emotional state of patients and their attenders. While we as humans are bound to have our emotional states and thoughts, empathy is a very important skill we must possess or develop. We believe that this does not come naturally to most, and that is nothing to be ashamed of. It must be incorporated into medical training and must be a constant reminder to us till it becomes a learned behaviour and habit. We must not take the reaction of the patient or attender personally, and rather be able to detach our expectation of their reaction and accept their emotional state.
Understanding	Medicine is a highly specialised science, that the general public has a poor understanding of. When someone does not understand something, they automatically tend to fear it. That fear of the unknown makes the individual also doubt what they are being told. It can also be the cause of people having unrealistic expectations which may be very different from the real scenario. This understanding of the situation we think goes beyond the educational status of an individual. While doctors can understand the status of the individual’s health and the problems by looking at the clinical findings and investigations reports, to the patient and attenders there are very few visual cues to go by. So, despite what is being communicated to the patient and attenders, they often fail to grasp the gravity of the situation or the importance of certain information conveyed to them.	Providing the correct treatment is not sufficient. Counselling of the patient and attenders is of utmost importance. Patients and attendees must be made aware of the situation and status throughout the treatment and not just when things go south. It is not necessary to make patients and attendees understand the technicalities of their medical condition, but we do need them to understand the gravity of the situation and treatment plans. Managing expectations is of paramount importance.
Finance	While healthcare in India has grown leaps and bounds in the last few decades with technological advancements, unfortunately, health insurance coverage is still poor, with a vast majority of patients having to pay out of their pockets for medical treatment. This, consciously or subconsciously, does play a role in the doctor-patient relationship and the expectations of the patients. The expenses incurred are very often a major distressing factor for the patient and their family, which may be expressed in the form of dissatisfaction with non-financial aspects of the treatment. All too often, the threat of violence is a ploy used by patients and attendees to obtain financial leeway. In several instances, patients have resorted to violence when they have been unable to pay hospital bills.	Doctors need to dissociate medical treatment and financial discussions. It is our strong recommendation, that doctors not discuss financial aspects of treatment with the patient and leave that to be done by administrative persons. We say that because the patients and attenders need to understand that doctors are not determining the costs of everything, and are not directly receiving shares of payments made at hospitals. This system of financial discussions being handled by someone other than the doctor is followed in many developed countries. Unfortunately in India due to a lack of manpower, doctors have to often wear both caps. But that is a recipe for disaster, as patients then hold the doctor responsible for financial aspects as well and patients attribute their financial difficulties to the doctor. This will eventually lead to them not trusting the doctor or being dissatisfied with medical decisions as well. Hence, whenever possible, we recommend insisting that the financial aspects of the treatment be communicated and discussed by someone other than the treating doctors.
Societal opinion on doctors	Many patients are prejudiced against doctors. Citing the cost of medical care, they assume doctors to be high-earning individuals. The general public fails to recognise that the majority of medical costs are for diagnostic tests, medications, and hospital fees, none of which the doctor receives. There is a false impression among large fractions of society that doctors and hospitals overcharge. This leads to a lack of trust. Oftentimes patients are not trusting of doctors and hospitals right from the offset. There are widespread beliefs that doctors and hospitals order unnecessary investigations and prescribe unnecessary treatments. This hostility and negativity make patients view every action of the doctor with an index of suspicion, and this leads to them blaming the doctor at the first sign of trouble or dissatisfaction.	It is not possible to individually change the societal opinion of doctors among the public. But considering the atmosphere of suspicion, doctors need to gain the trust and confidence of the patient by being transparent and communicating well with the patient. Discussions detailing the reasoning for investigations and treatment options recommended must be had. This may seem like a burden to doctors, who may argue that they make clinical decisions with the best intentions of the patient in mind, but that may not suffice. One must make the patient understand the reasoning behind the decisions and recommendations to ensure that the patient is cooperative rather than reluctant and suspicious.
Educational status	Unlike developed countries, in India, we may be dealing with patients with different education statuses. A higher education status should not be presumed to necessarily mean a better understanding of medical conditions and cooperation for treatment. While a higher educational status of the patient may make it easier to explain the medical condition, it can also lead to increased questions and doubts of the patients. They may also have various misconceptions arising out of misinformation obtained from unreliable sources on the internet. If these misconceptions are not dispelled, they will become a source of lack of trust in the doctor. The challenge on the other hand in dealing with patients of lower educational status may be in making them understand medical aspects of the condition and the advice given. While we must be unbiased in our commitment to our patients irrespective of educational qualifications and income status, if we have the same approach in dealing with patients with varied educational statuses, it can lead to problems.	We must not a have straightjacket approach to our communications with patients. It must be tailored to the educational status and understanding of the patient. More effort may have to be put into making patients with lower educational status understand the condition and treatment instructions. In patients with higher educational status, we must be ready to answer more questions, and also be ready to dispel some of the misconceptions or misinformation they may have.
Political factors	In the complex societal structures of India, unfortunately, even things such as political factors have been a factor leading to violence against doctors. This may be lesser in metropolitan cities but is more prevalent in smaller cities and towns of the country. ‘Political power’ is used, or the threat of it is used, to strong-arm doctors and hospitals. It is used to gain prioritised treatment and monetary concessions. This again becomes a source for discourse. Mob attacks on medical establishments have occurred in several places due to these very factors.	This is where the hospitals must ensure the safety of their workforce and provide for a safe work environment. There should be strict security arrangements at hospitals to ensure that mobs are not allowed to form within the hospital, that goons are not allowed, and that police intervention is obtained when the threat of such political blackmailing or violence occurs.
Frustration against the system	Many times doctors become the outlet for the frustration of patients and attenders against the system. As the primary point of contact for patients, doctors become the outlet to vent out frustrations on all aspects from nursing care, to administrative delays, long queues, and even housekeeping issues. There is a feeling of helplessness and frustration among the patients, and the doctors become the only audience for their dissatisfaction. Doctors are dismissive of such factors as they are out of their realm of control. Eventually, not having an outlet for their frustrations, doctors become the convenient targets for patients to express their frustration with verbal or physical altercations.	There must be grievance redressal systems in hospitals for patients to be able to express their dissatisfactions and complaints. Whether the complaints are addressed adequately or not, it is still beneficial to have a grievance redressal system as it allows them to be able to express their dissatisfaction, thereby preventing the frustration from accumulating and eventually erupting in the form of physical violence. Importantly, it segregates those administrative issues from the responsibilities of the doctor and the medical treatment of the patient for which the doctor is responsible.
The judicial system	The judicial system in the country is plagued with problems. The absence of proper legal recourse for patients to seek against doctors might be detrimental to doctors. While there are multiple legal avenues for patients to seek compensation from doctors, all legal proceedings are drawn out and take ages. This leads to certain patients or attendees taking things into their own hands and turning violent. Violence is often justified by these patients and attendees as their form of quick justice.	Doctors are in a precarious situation where we don’t seem to receive speedy justice in courts, have to bear through unfounded legal accusations, and nor does settling the matter out of court seem practical and safe if the patients and attenders are agitated and angry. However, if having to choose between the two, it is always better to fight the battle in court, with facts and information, rather than be tolerant of physical violence. If a disagreement with a patient or attenders does not seem to be coming to a civil conclusion, it is better to suggest the matter be dealt with within the court of law rather than let the discussion become abusive and violent.

Charting the course ahead

There is no easy, quick solution to the problem of violence against doctors. All of the factors we discussed are inherent problems in the doctor-patient relationship. While providing strict legal punishment for violence will go a long way in dissuading persons from resorting to violence, the factors we mentioned will still have to be addressed to avoid the situation where a patient or attendee considers resorting to violence. We believe understanding the factors at play and following the recommendations we made for each of those points would go a long way in preserving a healthy doctor-patient relationship and decreasing the violence against doctors.
